# Effect of massed versus distributed preclinical training on subgingival instrumentation skill across the preclinical-to-clinical transition

**DOI:** 10.1038/s41598-026-62235-6

**Published:** 2026-07-27

**Authors:** Laura Glevitzky, Miriam Cyris, Simone Sutor, Claudia Springer, Maren Kahl, Mohamed Mekhemar, Sonja Sälzer, Christian Graetz

**Affiliations:** Clinic of Conservative Dentistry and Periodontology, University Hospital Schleswig-Holstein, Kiel, Germany

**Keywords:** Scaling and root planing, Non-surgical periodontal therapy, Skill consolidation, Speed–accuracy trade-off, Digitised training, Dental curriculum, Diseases, Health care, Medical research

## Abstract

Consolidation of preclinical subgingival instrumentation skills during early clinical exposure is poorly quantified, especially after the new German dental licensing regulation (nGDLR). Within a shared digitised training programme (DTP), we compared in vitro performance after preclinical training and after the first periodontal patient course under distributed (oGDLR; 7th semester, S7) versus massed (nGDLR; 6th semester, S6) schedules. In a non-randomised, sequential two-cohort design (a consequence of a 2020 curriculum change), 102 students (oGDLR, distributed/longitudinal schedule, n = 45; nGDLR, massed/block schedule, n = 57) provided 136 evaluations after preclinical DTP (nGDLR S6, oGDLR S7) and/or after the first periodontal patient course (S8). Because oGDLR training (S7) overlapped with first clinical contacts whereas nGDLR training (S6) preceded intensive patient care by ~ 1.5 semesters, schedule density and clinical proximity co-vary and cannot be fully separated. Only 34 students (oGDLR n = 22; nGDLR n = 12) were evaluated at both time points, so most comparisons are cross-sectional. Each student instrumented six teeth with Gracey curettes (GRA) and a sonic scaler (AIR) on periodontitis manikins. Effectiveness of simulated plaque removal (ESP), treatment time, calculus removal and an 8-item Likert self-assessment were analysed by non-parametric tests and multivariable regression with cluster-robust SE. oGDLR was associated with higher ESP (β=+5.16%, p = 0.001) and twofold higher calculus removal (adjusted OR 2.00, p < 0.001); treatment time was comparable. From post-DTP to S8 both cohorts became faster by ~ 60s/tooth (p < 0.001) while ESP declined (β=−4.26%, p = 0.002) — a speed–accuracy trade-off; the schedule×time interaction was non-significant. In the paired subgroup, the trade-off was confirmed in oGDLR (n = 22; ESP − 7.2%, p < 0.001) but power-limited in nGDLR (n = 12). Excluding magnifying-loupe users (concentrated in nGDLR) widened rather than narrowed the oGDLR ESP advantage (β=+6.02%, p < 0.001). nGDLR rated AIR more favourably (learnability OR 0.40; effectiveness OR 0.48). Pass rates (≥ 55% ESP) exceeded 86%. In this non-randomised cohort, the distributed schedule was associated with higher absolute performance, but the decline occurred similarly under both schedules. Because schedule density could not be separated from proximity to clinical exposure, and because the data are largely cross-sectional and derive from a manikin-based in vitro model, these associations should not be read as causal. The transition to first patient care is vulnerable: cleaning quality may erode as students prioritise speed. Distributed preclinical practice was associated with a higher absolute baseline, but the decline across the transition was similar in both schedules; structured reinforcement during the early clinical phase remains necessary to consolidate competence.

## Introduction

The subgingival removal of biofilm and mineralised deposits is a cornerstone of non-surgical periodontal therapy (NSPT) and is recommended as step 2 by the EFP S3 guideline, irrespective of the instrument chosen (hand or power-driven)^1,2^. However, correct application of these instruments is among the most demanding fine-motor skills in dental education, sensitive to operator skill and consistently associated with substantial residual deposits in undergraduate hands^3–5^. Reliable acquisition of this competence during dental training – and its transfer to early patient care – is therefore essential for safe entry into clinical periodontal care. Previous work from our group has shown that a structured longitudinal training programme with weekly repetitions enables undergraduate students to reach high cleaning efficacies (> 70%) with both Gracey curettes (GRA) and sonic scalers (AIR)^6^, and that the digitised version of this programme (DTP) is superior to the former conventional programme^7^. Similar benefits have been described for multisensory and virtual-reality haptic training^8,9^.

Although previous studies have described individual elements of preclinical periodontology training, most of them quantify performance at a single time point immediately after preclinical training. The extent to which this competence consolidates during transition to clinical practice has rarely been examined prospectively. From a motor-learning perspective, this transition is critical. Forgetting processes (Ebbinghaus-type decay; Wang et al.^10^ compete with consolidation through new task experience, while the procedural time pressure of patient care may shift the speed–accuracy trade-off^11,12^ towards speed at the expense of precision. Schedule density (massed vs. distributed) is a well-established modifier of motor-skill retention^13–15^ and is therefore expected to determine how robust the baseline competence is at the time of clinical exposure. Recent work in surgical skills training has further shown that distributed schedules with short inter-session intervals are particularly effective^16^.

At Kiel University, the 2020 revision of the German dental licensing regulation (GDLR)^17^ prompted a shift from a longitudinal subgingival instrumentation training schedule (oGDLR; 9 h distributed across 12 weekly 45-min sessions during the 7th semester, running in parallel with the first patient contacts for conservative treatments and supportive periodontal-therapy (SPT) towards the end of that semester) to a condensed block format (nGDLR; 9 h delivered in three half-days within one single week of the 6th semester, separated from intensive periodontal patient treatment by approximately 1.5 clinical semesters in the 8th semester). Both schedules used the same validated DTP (the constant)^6^, allowing the effects of schedule density and its temporal proximity to clinical reinforcement (the modifier) to be evaluated on a common substrate. Both formats are also embedded in current educational frameworks for undergraduate periodontology^18–20^.

The aim of the present in vitro cohort study was therefore (i) to quantify how subgingival instrumentation competence evolves from the post-DTP evaluation (end of the 6th semester for nGDLR, S6; end of the 7th semester for oGDLR, S7) to the end of the first periodontal patient-treatment course (end of the 8th semester, S8), and (ii) to test whether preclinical schedule density and its temporal coupling to clinical exposure modify this consolidation trajectory. We hypothesised that distributed preclinical practice (oGDLR), blended with first clinical contacts, would yield higher cleaning efficacy and better calculus removal than the massed block format (nGDLR) at both time points, while the longitudinal change towards S8 — and any speed–accuracy re-weighting — would be comparable across schedules.

## Materials and methods

### Study design and ethics

This experimental in vitro cohort study with partial longitudinal measurements was conducted at the Clinic of Conservative Dentistry and Periodontology, University of Kiel, Germany, between October 2022 and July 2024. The study was approved by the Ethics Committee of the Medical Faculty of Kiel University (D509/18) and conducted in accordance with the Declaration of Helsinki and its later amendments. All participants provided written informed consent after receiving oral and written information before the first evaluation. Participation was voluntary, did not affect course grades, and complied with the STROBE statement^21^. Reporting of the training intervention followed the CReDECI principles for complex educational interventions. Reasons for non-participation were not formally collected.

### Participants and training concepts

Undergraduate dental students were invited immediately after their preclinical DTP training (post-DTP evaluation: nGDLR end of S6, oGDLR end of S7) and/or at the end of S8 (after the first NSPT patient-treatment course). Cohort assignment depended on matriculation cohort and the regulation in force during preclinical training. For nGDLR, the post-DTP evaluation preceded all clinical patient courses; the 7th semester comprised first conservative-dentistry contacts (only two SPT sessions), and the 8th semester comprised supervised treatment of several periodontitis patients per week. For oGDLR, the 7th-semester practical sessions overlapped towards the end of the semester with first SPT patient contacts (S7 captured a naturally blended preclinical–clinical exposure); the 8th semester in turn comprised intensive supervised NSPT.

**Fig. 1:**
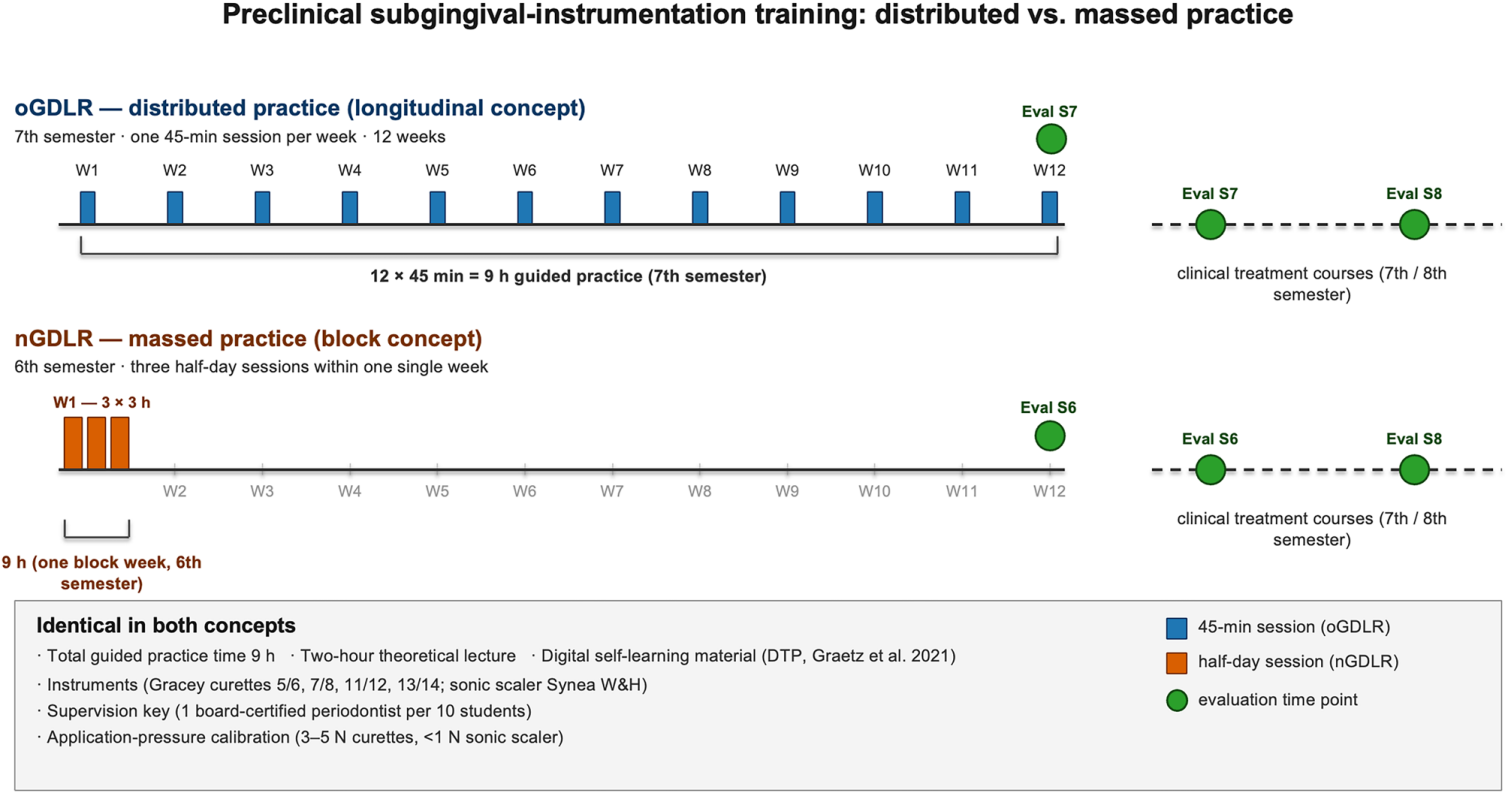
Schematic of the two preclinical training concepts and evaluation time points. Old licensing regulation (oGDLR): longitudinal distributed-practice concept with one weekly 45-min session over 12 weeks of the 7th semester (9 h), running in parallel with the first supportive periodontal-therapy patient contacts towards the end of that semester. New licensing regulation (nGDLR): compact block concept with three half-day sessions (3 h each, 9 h total) within one single week of the 6th semester, separated from intensive periodontal patient treatment by approximately 1.5 clinical semesters. Total guided practice time was comparable (9 h) in both concepts; the schedules differed both in temporal distribution and in temporal proximity to clinical reinforcement. Evaluations were performed at the end of the post-DTP semester (nGDLR S6; oGDLR S7) and/or at the end of the 8th semester (S8, after the first intensive periodontal patient-treatment course in the NSPT phase). For the nGDLR cohort, the 7th semester comprised first clinical contacts (mostly conservative dentistry, only occasional periodontal procedures); for the oGDLR cohort, the 7th semester combined preclinical practical training (DTP) with the first supportive periodontal-therapy patient contacts. The 8th semester comprised weekly periodontitis-patient treatment under supervision (NSPT phase) for both cohorts.

The longitudinal concept (oGDLR, distributed practice) comprised one 45-min practical session per week over 12 weeks of the 7th semester (9 h total). The block concept (nGDLR, massed practice) comprised three half-days within one week of the 6th semester (9 h total), entirely preceding clinical contact (Fig. 1). Both concepts shared the same theoretical introduction, digital self-learning material (DTP), instruments, supervision key (one board-certified periodontist per ten students) and calibration of application pressure (3–5 N for GRA, <1 N for AIR). Personal data (age, sex, handedness, previous medical/dental education, regular use of magnifying loupes) were collected pseudonymously. Each student received a unique identifier enabling longitudinal tracking. For clarity, the two schedules are also referred to by plain-language descriptors throughout: oGDLR as the distributed (longitudinal) schedule and nGDLR as the massed (block) schedule. Attendance at the preclinical training was mandatory in both curricula, and all participating students completed the full 9 h of guided practice (oGDLR 12 × 45 min; nGDLR 3 × 3 h); there were no partial-dose participants. The subsequent clinical courses (S7/S8) followed a standardised curricular structure with comparable patient-treatment requirements per student, although the exact number and difficulty of patients treated could not be individually standardised.

### Experimental setup and manikin heads

At each evaluation, participants instrumented six predefined test teeth (maxillary 11, 14, 16; mandibular 31, 34, 37) on four surfaces each (vestibular, oral, mesial, distal), yielding up to 24 surface-level observations per participant, instrument and time point. Instruments were Gracey curettes (GRA; shapes 5/6, 7/8, 11/12 and 13/14; American Eagle Instruments, Missoula, MT, USA) and a sonic scaler (AIR; Synea, W&H, Bürmoos, Austria) at “medium” amplitude with straight, right- and left-curved tips (1AP, 2APr, 2APl) and water cooling (30 ml/min). Instrument sequence was randomised per participant and jaw. Participants instrumented each tooth until they subjectively reached maximal removal (≤ 6 min/tooth). Curettes were inspected and sharpened by a trained dental hygienist before each test day.

Instrumentation was performed on identical manikin heads with modified periodontitis models (Frasaco, Tettnang, Germany; moderate to advanced horizontal bone loss and isolated deep vertical pockets, mean PPD 5.8 ± 2.1 mm, range 3–11 mm). Test teeth were coated with a thin transparent fluorescent varnish simulating biofilm and a modified commercial varnish (A-CK, Frasaco) simulating subgingival calculus; the reproducible coating and planimetric procedure has been described in detail previously^6,22^.

### Outcomes

The primary outcome was effectiveness of simulated plaque removal (ESP, cleaned area in % of total coated area). Secondary outcomes were treatment time per tooth (s) and successful removal of simulated subgingival hard deposits. Simulated calculus was applied to the approximal surfaces; a tooth could therefore carry calculus on more than one surface. Each calculus-bearing surface was classified as residual calculus present or successfully removed, and successful calculus removal for a given student and evaluation was defined as the proportion (%) of originally calculus-bearing surfaces from which the deposit was completely removed. The planimetric ESP evaluation and the calculus scoring were performed by a single examiner (L.G.) who was blinded to curriculum concept and to time point and worked on pseudonymised models; treatment time was recorded automatically by the software and was therefore independent of examiner judgement. No duplicate scoring of a subset was performed, so no study-specific intra- or inter-rater reproducibility coefficient is available; the planimetric/fluorescence procedure followed the standardised method described previously^6,22^. Self-assessment was obtained pseudonymously via an eight-item Likert questionnaire (1 = low/negative, 5 = high/positive): F1 learnability, F2 fatigue, F3 perceived time-saving, F4 substance preservation, F5 perceived effectiveness, F6 root-surface characteristics, F7 applicability of GRA, F8 applicability of AIR^6,7^. This questionnaire was not newly developed for the present study; it is the instrument used in our previous work on the same training programme^6,7^ and was applied here unchanged. It has not undergone separate formal psychometric validation, so the self-assessment results are interpreted descriptively. Root surface destruction and roughness were not assessed.

### Statistical analysis

Data were aggregated to the student level (mean per student, instrument and evaluation). Distribution was checked with Shapiro–Wilk; non-parametric tests were used throughout. Continuous variables are presented as mean ± SD, Likert items as median [IQR], categorical variables as n (%). Between- and within-group comparisons used Mann–Whitney U or Wilcoxon signed-rank tests. Multivariable linear regression (ESP, time) and logistic regression (calculus removal) with cluster-robust SE adjusted for curriculum concept, semester, sex, age, previous education and loupes; ordinal logistic regression was used for Likert items F1, F3 and F5. For interpretation, ESP results were additionally summarised against three pragmatic, a priori descriptive bands (pass ≥ 55%, high-level ≥ 70%, excellent ≥ 80%); these bands were chosen for descriptive clarity rather than as formally validated competence thresholds (see Limitations). The post-DTP-versus-S8 contrasts reported in Table 1 are unpaired comparisons of partly different students, because most students were evaluated at only one time point. To complement these, paired Wilcoxon signed-rank tests were performed in the subset of students evaluated at both time points (oGDLR *n* = 22; nGDLR *n* = 12). To address the imbalance in magnifying-loupe use, a sensitivity analysis repeated the ESP comparison and the adjusted ESP model after excluding all loupe users. No prospective power calculation was feasible; paired results in the small nGDLR subgroup (*n* = 12) are reported but interpreted with caution given limited power. Analyses used IBM SPSS v.30; two-sided *p* < 0.05.

**Table1:** Participants’ characteristics by curriculum concept.

Variable	nGDLR (*n* = 57)	oGDLR (*n* = 45)	Total (*n* = 102)
Students, n	57	45	102
Evaluations, n	69	67	136
Evaluations at 6th semester (nGDLR)/7th semester (oGDLR), n	55	37	92
Evaluations at 8th semester, n	14	30	44
Sex (female/male), n	42/15	37/8	79/23
Age (years), mean ± SD	24.40 ± 2.81	24.16 ± 2.95	24.29 ± 2.87
Previous medical/dental education (yes/no), n	18/39	10/35	28/74
Regular use of magnifying loupes (yes/no), n	26/31	2/43	28/74
Handedness (right/left), n	55/2	44/1	99/3

*oGDLR* old German dental licensing regulation (longitudinal training concept, distributed practice), *nGDLR* new German dental licensing regulation (block training concept, massed practice).

## Results

### Participants

**Fig. 2:**
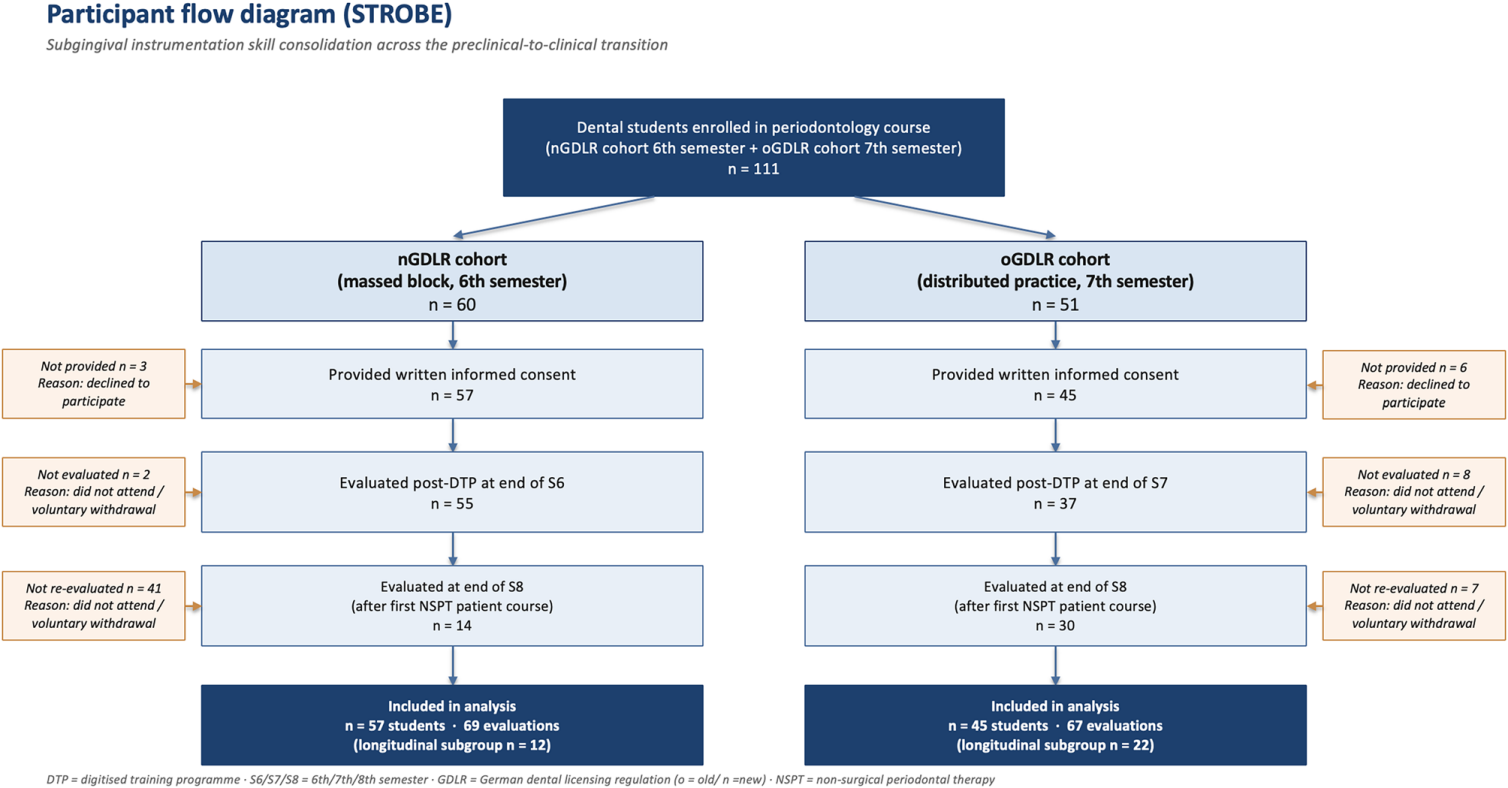
STROBE participant flow diagram. Boxes show the number of students evaluated at each stage; side callouts indicate the number and reason for non-evaluation. Longitudinal subgroup (students evaluated at both post-DTP and S8): nGDLR *n* = 12, oGDLR *n* = 22.

A total of 102 undergraduate dental students (79 female, 23 male; mean age 24.3 ± 2.9 years) contributed 136 evaluations (Fig. 2): 69 under nGDLR (55 at S6, 14 at S8) and 67 under oGDLR (37 at S7, 30 at S8). The two cohorts were comparable for age, sex and previous education, but the nGDLR cohort used magnifying loupes more frequently (26/57 vs. 2/45; Table 2). By study design, the post-DTP evaluation was scheduled at the end of the 6th semester for nGDLR but at the end of the 7th semester for oGDLR, reflecting the different placement of the practical training in the two regulations.

**Table 2:** Primary outcome (Effectiveness of simulated plaque removal (ESP), in %) and secondary outcomes (treatment time per tooth, s; successful calculus removal, %) by curriculum concept (oGDLR, distributed/longitudinal schedule; nGDLR, massed/block schedule) and semester. Sample sizes (n evaluations) are given in each column header. Mean ± SD; Mann–Whitney U test; significant p-values (*p* < 0.05) in bold.

Variable	nGDLR S6 (*n* = 55)	nGDLR S8 (*n* = 14)	*p* (S6 vs. S8)	oGDLR S7 (*n* = 37)	oGDLR S8 (*n* = 30)	*p* (S7 vs. S8)	*p* (nGDLR vs. oGDLR) post-DTP	*p* (nGDLR vs. oGDLR) S8
Overall ESP (%)	67.1 ± 8.9	63.5 ± 4.5	0.068	71.9 ± 6.7	66.0 ± 8.7	**0.004**	**0.024**	0.178
Overall time (s/tooth)	212.7 ± 37.9	132.9 ± 37.0	**< 0.001**	214.0 ± 37.2	162.3 ± 51.3	**< 0.001**	0.984	**0.049**
Overall calculus removal (%)	60.9 ± 18.1	54.8 ± 20.1	0.289	75.5 ± 20.2	66.1 ± 19.3	0.072	**< 0.001**	0.092
ESP (%) GRA	67.6 ± 10.4	63.9 ± 5.8	0.099	73.8 ± 5.7	66.3 ± 9.0	**< 0.001**	**0.005**	0.320
Time (s/tooth) GRA	229.2 ± 38.6	150.5 ± 38.4	**< 0.001**	231.3 ± 38.8	173.0 ± 55.2	**< 0.001**	0.959	0.186
Calculus removal (%) GRA	63.0 ± 25.4	52.4 ± 27.6	0.165	77.5 ± 24.9	70.0 ± 28.8	0.307	**0.004**	**0.050**
ESP (%) AIR	66.7 ± 9.0	63.1 ± 5.3	**0.039**	70.0 ± 8.8	65.7 ± 9.7	0.095	0.145	0.222
Time (s/tooth) AIR	196.1 ± 49.2	115.3 ± 42.5	**< 0.001**	196.7 ± 51.8	151.6 ± 58.2	**0.002**	0.946	**0.036**
Calculus removal (%) AIR	58.8 ± 22.6	57.1 ± 24.2	0.890	73.4 ± 22.7	62.2 ± 17.5	**0.023**	**0.004**	0.712

GRA Gracey curettes, *AIR* sonic scaler, *oGDLR* old German dental licensing regulation (longitudinal training concept, distributed practice), *nGDLR* new German dental licensing regulation (block training concept, massed practice).

### Effectiveness of simulated plaque removal (ESP)

ESP was significantly higher under oGDLR than nGDLR at the post-DTP evaluation (oGDLR S7: 71.9 ± 6.7% vs. nGDLR S6: 67.1 ± 8.9%; *p* = 0.024). No significant difference was observed at S8 (66.0 ± 8.7% vs. 63.5 ± 4.5%; *p* = 0.178). Within oGDLR, ESP decreased significantly from S7 to S8 (*p* = 0.004), whereas only a non-significant trend was seen in the nGDLR cohort from S6 to S8 (*p* = 0.068; Table 2). After adjustment, curriculum concept remained an independent predictor of ESP (oGDLR vs. nGDLR: β = +5.16%; 95% CI 2.24–8.09; *p* = 0.001), together with a small negative semester effect (β = − 4.26%; *p* = 0.002; Table 3). None of the other covariates reached significance in the overall model; in an exploratory sub-cohort regression of the nGDLR group, however, regular use of magnifying loupes was independently associated with + 7.25% ESP (95% CI 2.76–11.73; *p* = 0.002), whereas in the oGDLR sub-cohort loupes could not be modelled (only *n* = 2 users). In the sensitivity analysis excluding all loupe users, the oGDLR advantage in ESP did not diminish but increased: at the post-DTP evaluation ESP was 72.0 ± 6.9% (oGDLR, *n* = 35) versus 63.7 ± 8.1% (nGDLR, *n* = 29; *p* < 0.001), and the adjusted concept effect rose to β = +6.02% (95% CI 2.96–9.08; *p* < 0.001). Because loupe use was concentrated in the nGDLR cohort, it partly masked rather than created the oGDLR advantage.

**Table 3:** Instrument comparison (GRA vs. AIR) by curriculum concept (oGDLR, distributed/longitudinal schedule; nGDLR, massed/block schedule) and semester, pooled at the student level. Mean ± SD; Mann–Whitney U test. Significant p-values (*p* < 0.05) in bold.

Group	Outcome	GRA	AIR	*p*
nGDLR S6 (*n* = 55)	ESP (%)	67.6 ± 10.4	66.7 ± 9.0	0.645
	Time (s/tooth)	229.2 ± 38.6	196.1 ± 49.2	**< 0.001**
	Calculus removal (%)	63.0 ± 25.4	58.8 ± 22.6	0.329
nGDLR S8 (*n* = 14)	ESP (%)	63.9 ± 5.8	63.1 ± 5.3	0.765
	Time (s/tooth)	150.5 ± 38.4	115.3 ± 42.5	**0.016**
	Calculus removal (%)	52.4 ± 27.6	57.1 ± 24.2	0.705
oGDLR S7 (*n* = 37)	ESP (%)	73.8 ± 5.7	70.0 ± 8.8	**0.039**
	Time (s/tooth)	231.3 ± 38.8	196.7 ± 51.8	**0.005**
	Calculus removal (%)	77.5 ± 24.9	73.4 ± 22.7	0.310
oGDLR S8 (*n* = 30)	ESP (%)	66.3 ± 9.0	65.7 ± 9.7	0.935
	Time (s/tooth)	173.0 ± 55.2	151.6 ± 58.2	0.152
	Calculus removal (%)	70.0 ± 28.8	62.2 ± 17.5	0.114

*GRA* Gracey curettes, *AIR* sonic scaler, *oGDLR* old German dental licensing regulation (longitudinal training concept, distributed practice), *nGDLR* new German dental licensing regulation (block training concept, massed practice).

The pass rate (≥ 55% ESP) was ≥ 86% across all groups (87.3%, 92.9%, 97.3%, 86.7% for nGDLR S6, nGDLR S8, oGDLR S7, oGDLR S8 respectively). High-level performance (≥ 70% ESP) was achieved by 70.3% of oGDLR S7 students versus 43.6% under nGDLR S6, and decreased in both concepts to 40.0% and 7.1% respectively at S8. Excellent performance (≥ 80% ESP) remained rare (0–10.8%).

### Treatment time

Treatment time did not differ between groups at the post-DTP evaluation (nGDLR S6: 212.7 ± 37.9 s/tooth vs. oGDLR S7: 214.0 ± 37.2 s/tooth; *p* = 0.984). From the post-DTP evaluation to S8 treatment time decreased by approximately 50–80 s/tooth in both cohorts (both *p* < 0.001; Table 2). In the adjusted model (Table 3) semester was by far the strongest predictor (β = − 59.46 s/tooth; *p* < 0.001), whereas the curriculum concept had no significant effect (β = +6.18 s/tooth; *p* = 0.444). Age was associated with a small reduction of treatment time (β = − 3.15 s/year; *p* = 0.028).

### Removal of simulated hard deposits

Successful calculus removal was substantially higher under oGDLR than under nGDLR at the post-DTP evaluation (oGDLR S7 75.5 ± 20.2% vs. nGDLR S6 60.9 ± 18.1%, *p* < 0.001). At S8 both cohorts had lower calculus removal (oGDLR S8 66.1 ± 19.3% vs. nGDLR S8 54.8 ± 20.1%), and the unadjusted between-group difference was no longer statistically significant (*p* = 0.092; Table 2). The adjusted model confirmed the curriculum concept as the strongest predictor (oGDLR vs. nGDLR: OR 2.00; 95% CI 1.39–2.88; *p* < 0.001) and showed a marginal semester effect (OR 0.73; *p* = 0.049; Table 3).

### Instrument comparison (GRA vs. AIR)

Pooled across all 136 evaluations, GRA showed numerically higher ESP (68.6% vs. 67.0%; *p* = 0.124) and higher calculus removal (67.4% vs. 63.4%; *p* = 0.084) than AIR, though neither difference reached significance; the direction was consistent in three of four strata for calculus removal and all four for ESP, but stratum-specific significance was inconsistent (Table 4). AIR was consistently faster (about 20–35 s/tooth; pooled mean 178.1 vs. 209.3 s/tooth, *p* < 0.001), and this was the only instrument-related difference that remained robust across all strata.

**Table 4:** Multivariable regression models for the three objective outcomes. Linear regression for ESP and time; logistic regression for successful calculus removal. Cluster-robust standard errors; 95% CI; significant p-values (p<0.05) in bold.

Variable	ESP (%) β (95% CI)	*p*	Time (s/tooth) β (95% CI)	*p*	Calculus removal OR (95% CI)	*p*
Curriculum concept (oGDLR vs. nGDLR)	5.16 (2.24; 8.09)	**0.001**	6.18 (–9.66; 22.02)	0.444	2.00 (1.39; 2.88)	**< 0.001**
Semester (S8 vs. post-DTP)	–4.26 (–6.95; − 1.58)	**0.002**	–59.46 (–71.72; − 47.20)	**< 0.001**	0.73 (0.54; 1.00)	**0.049**
Sex (female vs. male)	–1.97 (–5.64; 1.71)	0.294	–1.69 (–18.70; 15.33)	0.846	0.84 (0.58; 1.23)	0.381
Age (per year)	–0.43 (–1.08; 0.21)	0.188	–3.15 (–5.96; − 0.35)	**0.028**	0.95 (0.90; 1.01)	0.087
Previous education (yes vs. no)	–0.34 (–3.63; 2.96)	0.842	–0.40 (–17.67; 16.87)	0.964	0.97 (0.66; 1.43)	0.888
Magnifying loupes (yes vs. no)	3.59 (–0.28; 7.46)	0.069	–5.07 (–22.23; 12.10)	0.563	1.10 (0.73; 1.66)	0.652

*GRA* Gracey curettes, *AIR* sonic scaler, *ESP* Effectiveness of simulated plaque removal, *oGDLR* old German dental licensing regulation (longitudinal training concept, distributed practice), *nGDLR* new German dental licensing regulation (block training concept, massed practice).

### Interindividual longitudinal changes (post-DTP → S8)

In students evaluated at both time points (oGDLR *n* = 22; nGDLR *n* = 12), treatment time improved (decreased) in 91% of students in both cohorts (oGDLR 90.9%; nGDLR 91.7%), whereas ESP decreased in about 80% (oGDLR 81.8%; nGDLR 75.0%) and successful calculus removal decreased in about 60% (oGDLR 59.1%; nGDLR 58.3%). In paired Wilcoxon signed-rank tests within this subgroup, the oGDLR cohort (*n* = 22) showed a statistically significant trade-off, with ESP decreasing from 72.7 to 65.5% (*p* < 0.001) and treatment time decreasing by ~ 53 s/tooth (*p* < 0.001). In the nGDLR cohort (*n* = 12), treatment time also decreased significantly (~ 74 s/tooth, *p* = 0.001), whereas the ESP change (− 4.8%) did not reach significance (*p* = 0.13); given the small number of pairs, this should be read as limited statistical power rather than absence of a trade-off. Paired changes in calculus removal did not reach significance in either subgroup (both *p* > 0.19). Overall, the descriptive pattern is consistent with a speed–accuracy trade-off in both concepts.

### Self-assessment (questionnaire)

At the post-DTP evaluation, nGDLR students rated learnability of AIR (F1: median 4[4–4] vs. 4[3–4]; *p* = 0.024) and its perceived effectiveness (F5: 4[3–4] vs. 3[2–4]; *p* = 0.016) more favourably than oGDLR students. No significant differences were found for items F2, F3, F4, F6, F7 or F8 or between the post-DTP evaluation and S8 within either cohort. After adjustment, the curriculum concept remained the only significant covariate: oGDLR students had a lower probability of a more favourable rating for F1 (OR 0.40; 95% CI 0.17–0.97; *p* = 0.042) and F5 (OR 0.48; 95% CI 0.23–1.00; *p* = 0.049; Table 5). After appropriately accounting for the difference between machine-based and manual instrumentation, no correlation was found between the subjective assessment of effectiveness (F5) and the objectively measured difference in biofilm removal in either cohort. In contrast, perceived time efficiency (F3) showed a significant correlation with the actual difference in treatment time (nGDLR: ρ = −0.30, *p* = 0.013; oGDLR: ρ = −0.28, *p* = 0.020).

**Table 5:** Self-assessment of the power-driven (sonic scaler) instrumentation relative to Gracey curettes (5-point Likert items F1–F8; 1 = low/negative, 5 = high/positive). Left: descriptive data (median [IQR]) and between-concept comparisons by Mann–Whitney U test. Right: adjusted ordinal logistic regression (proportional-odds) for the three hypothesis-driven items F1, F3 and F5; curriculum concept oGDLR vs. nGDLR; further covariates (semester, sex, age, previous education, magnifying loupes) not shown (all *p* > 0.10 for F1/F3/F5). OR > 1 = higher probability of a more favourable rating. Sample sizes (n evaluations) are given in each column header. Significant p-values (*p* < 0.05) in bold.

Item	nGDLR S6 (*n* = 55)	nGDLR S8 (*n* = 14)	oGDLR S7 (*n* = 37)	oGDLR S8 (*n* = 30)	*p* (nGDLR vs. oGDLR) post-DTP	*p* (nGDLR vs. oGDLR) S8	Adjusted OR oGDLR vs. nGDLR (95% CI)	*p*
F1 Learnability	4 [4–4]	4 [4–4]	4 [3–4]	4 [3–4]	**0.024**	0.141	0.40 (0.17; 0.97)	**0.042**
F2 Fatigue	4 [4–5]	4.5 [4–5]	4 [4–4]	4 [4–5]	0.084	0.321	–	–
F3 Perceived time-saving	4 [3–4]	4 [3–4]	4 [3–4]	3 [3–4]	0.717	0.489	1.00 (0.44; 2.23)	0.991
F4 Substance preservation	3 [3–4]	4 [3–4]	3 [3–4]	3 [3–4]	0.228	0.876	–	–
F5 Perceived effectiveness	4 [3–4]	3 [3–4]	3 [2–4]	3 [2–4]	**0.016**	0.486	0.48 (0.23; 1.00)	**0.049**
F6 Root-surface characteristics	3 [3–4]	3 [3–4]	3 [3–4]	3 [3–4]	0.107	0.989	–	–
F7 Applicability of GRA	4 [4–4]	4 [3–4]	4 [4–4]	4 [4–5]	0.849	0.163	–	–
F8 Applicability of AIR	4 [4–4]	4 [3–4]	4 [3–4]	4 [3–4]	0.227	0.734	–	

IQR, interquartile range; –, item not included in the adjusted regression (non-primary outcome); oGDLR, old German dental licensing regulation (longitudinal training concept, distributed practice); nGDLR, new German dental licensing regulation (block training concept, massed practice).

## Discussion

This in vitro cohort study tracked subgingival instrumentation across the preclinical-to-clinical transition under a unified digitised programme^6^, testing how schedule density and its temporal coupling to clinical exposure modify skill consolidation. The principal finding is a clear speed–accuracy trade-off: students became ~ 60s/tooth faster at S8 than at the post-DTP evaluation, while ESP and calculus removal declined by 4–9% points. Schedule density did not change this trajectory but was associated with higher absolute performance: distributed preclinical practice (oGDLR) was associated with higher ESP (β=+5.16%; *p* = 0.001) and twofold odds of complete calculus removal (OR 2.00; *p* < 0.001) versus the massed block (nGDLR), despite identical net guided-practice time. Pass rates (≥ 55% ESP) exceeded 86% in all strata, but high-level performance (≥ 70%) was less frequent under nGDLR.

These findings align with meta-analytic evidence that distributed practice supports motor and health-professions skill retention^13–15^, including a recent surgical-skills meta-analysis^16^. Spaced practice supports consolidation and somatosensory–motor integration, whereas massed training decays in line with the Ebbinghaus forgetting curve^10^. The larger effect on calculus than biofilm removal is plausible because tactile detection of subgingival hard deposits relies on a refined somatosensory feedback loop^23,24^, whereas treatment time was driven primarily by clinical experience — consistent with Oh et al.^25^. The transition itself produced a classic speed–accuracy trade-off^11,12^: increasing fluency and patient-care time pressure recalibrate operators towards speed before consolidation can stabilise quality. The non-significant schedule×time interaction indicates that this challenge is intrinsic to the transition rather than peculiar to one format. Pure forgetting cannot explain the concurrent gain in speed^10^.

Organisationally, the block format requires fewer resources and facilitates rotation of larger cohorts, aligning with modular and digitally blended teaching^26,27^, but may compromise long-term retention. If the aim is summative-threshold passing, the block concept appears sufficient; if the aim is robust fine-motor competence, distributed practice is superior. A pragmatic hybrid combining the compact block with short, distributed booster sessions during subsequent clinical semesters is supported by current frameworks^18,19^. An important caveat is that oGDLR practical sessions overlapped with the first SPT contacts (so S7 captured a naturally blended exposure) whereas nGDLR experienced a 1.5-semester gap before intensive NSPT; schedule density and proximity to clinical reinforcement therefore cannot be fully disentangled.

The instrument comparison showed a robust, instrument-inherent speed advantage for AIR (~ 20–35 s/tooth faster than GRA across all strata), consistent with our previous manikin-head studies^6,7,22^ and Suvan et al.^2^. GRA showed a numerically higher ESP and calculus removal in most strata, but these differences did not reach significance pooled or in most individual strata, so they should be interpreted as a trend rather than an established instrument-inherent effect.

The substantially higher use of magnifying loupes in nGDLR (26/57 vs. 2/45) is a notable ancillary finding. Within the nGDLR sub-cohort, regular loupe use was independently associated with + 7.25% ESP (*p* = 0.002), almost neutralising the concept-related disadvantage. This is consistent with our previous work^28,29^ and with parallel evidence from cavity preparation and endodontics^30–33^. A prospective controlled evaluation as standard nGDLR component is therefore warranted.

nGDLR students rated AIR more favourably for learnability and effectiveness despite lower objective performance — concordant with previous reports^6,7^. Less well-trained operators tend to overestimate themselves^34^, and self-reliant formats foster more realistic self-assessment^7^; this is supported by the absent correlation between perceived and measured effectiveness, while perceived time efficiency correlated with actual treatment time.

The main strength is the within-institution comparison under otherwise identical conditions^6,7,22^. Limitations include (i) the non-randomised sequential-cohort design due to the regulatory change; (ii) the partial longitudinal design (only a subset evaluated twice); (iii) the in vitro setting^5^; (iv) absence of root-surface destruction/roughness assessment^22^; (v) unequal use of magnifying loupes, although accounted for in regression and in a dedicated sensitivity analysis, which indicated the imbalance worked against rather than in favour of the main finding; and (vi) the asymmetric placement of preclinical training, so that schedule density and proximity to clinical reinforcement cannot be fully separated. Further limitations are that the ESP performance bands (≥ 55/70/80%) were pragmatic, a priori descriptive cut-offs and were not formally validated; that outcome scoring was performed by a single (blinded) examiner without duplicate assessment, so no study-specific intra-/inter-rater reproducibility coefficient is available; and that, because study participation and re-evaluation at S8 were voluntary, the students re-evaluated (particularly the smaller nGDLR S8 group) may not be fully representative, so attrition may bias the longitudinal estimates in an undetermined direction. As an in vitro manikin model with simulated biofilm and calculus, the study cannot capture access difficulty, bleeding, patient movement or anatomical variation, and its findings should not be assumed to translate directly to clinical patient outcomes.

## Conclusion

In this non-randomised cohort, the distributed schedule (oGDLR) was associated with higher absolute baseline performance than the massed block format (nGDLR), but the decline across the transition occurred similarly under both schedules. Because of the non-randomised sequential-cohort design, the largely cross-sectional (partial longitudinal) data, the magnifying-loupe imbalance, the inseparability of schedule density from clinical proximity, and the in vitro setting, no definitive causal conclusion about the superiority of either schedule can be drawn. The practical implication—that short, distributed booster sessions during the early clinical phase might help consolidate competence—should be regarded as a hypothesis for a future controlled study.

## Data Availability

The datasets generated and analysed during the current study are not publicly available due to national data protection law but are available from the corresponding author on reasonable request.

## Abbreviations

oGDLR: old German dental licensing regulation (longitudinal training concept, distributed practice)
nGDLR: new German dental licensing regulation (block training concept, massed practice)
GRA: Gracey curettes
AIR: Sonic scaler
ESP: Effectiveness of simulated plaque removal
DTP: Digitised training programme
SPT: Supportive periodontal therapy
NSPT: Non-surgical periodontal therapy
EFP: European federation of periodontology
OR: Odds ratio
CI: Confidence interval
SD: Standard deviation
IQR: Interquartile range
